# Effect of a prebiotic supplement on knee joint function, gut microbiota, and inflammation in adults with co-morbid obesity and knee osteoarthritis: study protocol for a randomized controlled trial

**DOI:** 10.1186/s13063-021-05212-w

**Published:** 2021-04-07

**Authors:** Rafael Fortuna, David A. Hart, Keith A. Sharkey, Rachel A. Schachar, Kelly Johnston, Raylene A. Reimer

**Affiliations:** 1 Human Performance Laboratory, Faculty of Kinesiology, University of Calgary, Calgary, Alberta Canada; 2 McCaig Institute for Bone and Joint Health, Department of Surgery, and Faculty of Kinesiology, University of Calgary, Calgary, Alberta Canada; 3 Hotchkiss Brain Institute and Snyder Institute for Chronic Diseases, Department of Physiology and Pharmacology, Cumming School of Medicine, University of Calgary, Calgary, Alberta Canada; 4 Rocky Mountain Health Clinic, Calgary, Alberta Canada; 5 Division of Hip and Knee Reconstruction, Department of Surgery, Cumming School of Medicine, University of Calgary, Calgary, Alberta Canada; 6 Faculty of Kinesiology and Department of Biochemistry and Molecular Biology, Cumming School of Medicine, University of Calgary, 2500 University Dr. NW, Calgary, Alberta Canada

**Keywords:** Knee osteoarthritis, Gut microbiota, Knee strength, Obesity, Knee joint pain, Prebiotic, Oligofructose-enriched inulin

## Abstract

**Background:**

Osteoarthritis (OA) is a chronic and painful condition where the articular cartilage surfaces progressively degenerate, resulting in loss of function and progressive disability. Obesity is a primary risk factor for the development and progression of knee OA, defined as the “metabolic OA” phenotype. Metabolic OA is associated with increased fat deposits that release inflammatory cytokines/adipokines, thereby resulting in systemic inflammation which can contribute to cartilage degeneration. There is currently no cure for OA. Prebiotics are a type of dietary fiber that can positively influence gut microbiota thereby reducing systemic inflammation and offering protection of joint integrity in rodents. However, no human clinical trials have tested the effects of prebiotics in adults with obesity suffering from knee OA. Therefore, the purpose of this double-blind, placebo-controlled, randomized trial is to determine if prebiotic supplementation can, through positive changes in the gut microbiota, improve knee function and physical performance in adults with obesity and knee OA.

**Methods:**

Adults (*n* = 60) with co-morbid obesity (BMI > 30 kg/m^2^) and knee OA (Kellgren-Lawrence grade II–III) will be recruited from the Alberta Hip and Knee Clinic and the Rocky Mountain Health Clinic and surrounding community of Calgary, Canada, and randomized (stratified by sex, BMI, and age) to prebiotic (oligofructose-enriched inulin; 16 g/day) or a calorie-matched placebo (maltodextrin) for 6 months. Anthropometrics, performance-based tests, knee pain, serum inflammatory markers and metabolomics, quality of life, and gut microbiota will be assessed at baseline, 3 months, 6 months (end of prebiotic supplementation), and 3 months following the end of the prebiotic supplementation.

**Clinical significance:**

There is growing pressure on health care systems for aggressive OA treatment such as total joint replacement. Less aggressive, yet effective, conservative treatment options have the potential to address the growing prevalence of co-morbid obesity and knee OA by delaying the need for joint replacement or ideally preventing its need altogether. The results of this clinical trial will provide the first evidence regarding the efficacy of prebiotic supplementation on knee joint function and pain in adults with obesity and knee OA. If successful, the results may provide a simple, safe, and easy to adhere to intervention to reduce knee joint pain and improve the quality of life of adults with co-morbid knee OA and obesity.

**Trial registration:**

Clinical Trials.gov NCT04172688. Registered on 21 November 2019.

## Background

Osteoarthritis (OA) is a chronic joint condition where the articular cartilage surfaces progressively degenerate, resulting in loss of joint range of motion, decline in function, progressive disability, pain, and reduced quality of life [1, 2]. Given its role in ambulation, knee OA is of particular concern and can lead to decreased quality of life, dependency, depression, and early death [3]. The treatment of OA is costly with an estimated $27 billion in direct and indirect costs annually in Canada [3]. A substantial portion of costs are attributed to loss of working days and the costs to cover aggressive surgical interventions such as a total knee replacement [3–5].

It is now well established that obesity is a primary risk factor for both the incidence and progression of OA [6–8]. OA linked to obesity has been defined as a separate “metabolic OA” phenotype [9, 10], representing 60% of the OA population [11]. Metabolic OA is associated with increased fat deposits that can release inflammatory cytokines/adipokines, thereby resulting in low-level systemic and local inflammation, which contributes to cartilage degeneration [12–14]. Currently, there is no cure or disease modifying treatment for OA, and the co-occurrence of knee OA and obesity accentuates the challenges associated with adherence to exercise interventions aimed at reducing body weight, improving joint function, and alleviating knee joint pain. However, obesity is considered a modifiable risk factor [8, 15] and diet interventions that target metabolic health may offer a window of opportunity to reduce systemic inflammation, thereby reducing knee joint pain and enabling participants to engage in exercise interventions to improve knee joint function.

Prebiotics are defined as a substrate that is selectively utilized by host microorganisms conferring a health benefit [16]. Inulin and/or oligofructose are two chicory root-derived prebiotics that are fermented in the gut and promote the growth of beneficial bacteria including *Bifidobacterium* [16]. The fermentation of prebiotics by intestinal bacteria results in the production of short-chain fatty acids (SCFA) which in turn can act as signaling molecules to influence host metabolism, immunity, and inflammation [17]. Part of the protection provided by SCFA is plausibly linked to their importance, particularly butyrate, in maintaining the epithelial barrier and preventing the translocation of bacterial proinflammatory molecules (e.g., lipopolysaccharide) across the gut wall [18, 19]. In clinical studies with individuals with overweight or obesity, prebiotics have been shown to reduce inflammation [20], improve glucose homeostasis [21–23], reduce body fat [24, 25], and improve appetite control [26, 27].

Rios et al. [28] and Schott et al. [29] recently showed that prebiotics can, through positive changes in gut microbiota, reduce systemic inflammation and offer protection to joint articular cartilage in rodent models of knee OA. More specifically, Rios et al. [28] showed that despite the intake of a high-fat/high-sucrose (HFS) diet, rats supplemented with the prebiotic oligofructose had significantly reduced knee joint damage, microbial dysbiosis, endotoxin levels, and insulin resistance compared to those consuming the HFS diet alone. Importantly, all of the indicated outcome measures were restored to levels observed in healthy lean chow-fed control animals [28]. Findings from this pre-clinical model suggest that obesity-related metabolic dysregulation and microbial dysbiosis play a role in the progression of metabolic OA. Modulating dysbiosis and metabolic dysfunction with prebiotic supplementation might protect against the development of knee joint damage. Similarly, Schott et al. [29] reported that obese mice fed prebiotic oligofructose were completely rescued from the joint degeneration seen in untreated obese mice and this was accompanied by reduced synovial inflammation and proinflammatory serum cytokines alongside a 1000-fold increase in the abundance of the beneficial bacterium *Bifidobacterium pseudolongum* and reduced abundance of proinflammatory bacteria *Peptostreptococcaceae* sp. and *Peptococcaceae rc*4-4 sp [29]. Despite this promising pre-clinical evidence for the protective effect of prebiotics on knee joint integrity, there are no human clinical trials in which the effects of prebiotics have been studied in adults with obesity also suffering from knee OA which represent a significant subset of idiopathic OA patients.

Prebiotics are a potentially innovative, inexpensive, and easy to adhere to intervention that could be used for the conservative management of OA patients. The aim of this study is to examine if prebiotic oligofructose-enriched inulin reduces knee joint pain and improves knee function, thereby improving quality of life through beneficial changes in gut microbiota and the reduction of systemic inflammation.

### Objectives

#### Primary objective

The primary objective of the present study is to determine if a 6-month prebiotic dietary intervention can improve knee joint function and physical performance in adults with obesity and knee osteoarthritis.

#### Secondary objectives

The secondary objectives are to determine if a 6-month prebiotic intervention can reduce knee pain, alter body composition (fat mass and lean mass), and improve quality of life. A mechanistic understanding of the potential changes will be investigated by examining changes in gut microbiota, serum inflammatory, and metabolomic markers in participants treated with the prebiotic supplement or placebo.

We hypothesize that prebiotic oligofructose-enriched inulin will reduce systemic inflammation, thereby alleviating knee joint pain and improving knee joint function. The intervention would be successful if it reduced knee joint pain sufficiently to enable participants to engage in a future exercise intervention with the goal to further increase knee joint function and reduce body fat, and thereby delaying/preventing the need for aggressive surgical intervention such as knee joint replacement.

## Methodology

### Ethics, consent, and permission

The present study has been approved (REB17-2363) by the Conjoint Health Research Ethics Board of the University of Calgary (Calgary, AB, Canada). All protocol modifications will be reviewed and approved by the ethics board. Voluntary, written, informed consent will be obtained from each participant. The trial has been registered at www.ClinicalTrial.gov (NCT04172688).

### Design, participants, and setting

The study is a single-center (all testing and visits occurring at the Human Performance Laboratory, University of Calgary, Calgary, Alberta, Canada), double blind, placebo-controlled randomized trial in which 60 adults (age 30–75 years) with obesity (body mass index (BMI) > 30 kg/m^2^) will be randomly assigned for six (6) months to either a placebo (*n* = 30; 6.6 g/day maltodextrin) or prebiotic (*n* = 30; 16 g/day oligofructose-enriched inulin) group. The prebiotic dosage selected for the present study is based on the dosage commonly used in previous studies with inulin and/or oligofructose that targeted metabolic outcomes including weight loss, adiposity, glycemia, and inflammatory markers [24–26, 30–33]. The age range was selected based on the increasing incidence rates for knee osteoarthritis that occur from the approximate age of 30 upwards [34]. We limited the upper end of the age range to 75 years given the known shift in gut microbiota composition that occurs with aging [35] that could potentially confound the study outcomes. Participants will visit the Human Performance Laboratory at baseline, 3, 6, and 9 months. At each visit, data will be collected for anthropometrics, knee function, knee pain, stool and blood samples, and questionnaires to monitor daily activities, food intake, and quality of life.

Participants will be recruited from the Alberta Hip and Knee Clinic and Rocky Mountain Health Clinic both located in Calgary, Alberta, Canada. Additionally, recruitment posters will be displayed on public notice boards and social media venues will be used to highlight the objectives of the study so that potential participants from the community in Calgary and surrounding area can contact the research team for more information. Potential participants will be screened for concomitant knee osteoarthritis and BMI > 30 kg/m^2^ and assessed for eligibility as per inclusion and exclusion criteria listed below.

### Randomization

Following confirmation from a medical doctor of a proper diagnosis of OA that fits the study criteria and alignment with the inclusion/exclusion criteria, stratified randomization of participants will occur using computer generated random numbers with an allocation ratio of 1:1 for placebo or the prebiotic intervention. The factors to be used for stratification are sex, BMI, and age. An investigator not involved in conducting the study will generate the allocation sequence and allocation (sequentially numbered) will only take place once the participant has consented to the study. The clinical trial coordinator will enroll and assign participants to the intervention groups. Participants, research staff, and those performing analysis will be blinded to the treatments and the prebiotic and placebo provided in identical opaque containers. At the end of the trial, participants will be asked to which group they believed they were assigned to assess the success of the blinding.

### Inclusion and exclusion criteria

Participants will be identified as eligible if they are male and female adults between 30 and 75 years of age, have a BMI > 30 kg/m^2^, and have a diagnosis of unilateral or bilateral knee osteoarthritis grades 2–3 according to the Kellgren-Lawrence radiographic rating scale. Exclusion criteria includes any previous knee joint trauma, knee surgeries, and corticosteroid injections; concomitant use of any weight loss medication or herbal weight loss products; previous bariatric or other intestinal surgery known to affect food intake or digestive function; presence of active infection, pregnancy, or lactation; regular use of a probiotic or prebiotic supplement within 3 months prior to enrollment; antibiotic use within 3 months to enrollment; weight loss > 3 kg within the preceding 3 months to enrollment; and uncontrolled cardiovascular or respiratory disease, active malignancy, or chronic infections. Eligibility will be assessed by the trial coordinator during a phone interview using a screening questionnaire.

### Prebiotic and placebo products

Participants will be randomized to receive either prebiotic (oligofructose-enriched inulin; Synergy1, Beneo, Mannheim, Germany; 16 g/day) or an isocaloric matched placebo (maltodextrin; Agenamalt 20.222, Agrana Starch, Konstanz, Germany; 6.6 g/day). In Canada, inulin and oligofructose are regulated as food ingredients and have been used extensively in clinical trials in adults and children across a wide variety of health conditions [16]. The prebiotic and placebo are both white powders that dissolve completely in water. The study products will be provided to participants in identical opaque containers. Participants will be instructed to mix the daily dose of study product in a glass of water and consume it at their convenience with advice to consume it later in the day to minimize any daytime gastrointestinal discomfort that could arise. Given that an increase in fermentative substrate in the gut can increase flatulence, participants will be instructed to consume half of the daily dose for the initial 2 weeks in order to minimize gastrointestinal discomfort. Following the first 2 weeks, participants will consume the full daily dose for the remainder of the 6-month intervention portion of the study. At the end of the 6 months, the participants will discontinue the study products and a 3-month wash-out period will occur. This study is designed to examine the effects of prebiotic independent of any other diet or exercise intervention; therefore, subjects will be encouraged to maintain their regular lifestyle, to eat until comfortably full and not to consciously try to gain or lose weight throughout the study. Routine individual management of knee osteoarthritis that is in place at the beginning of the trial will continue throughout the duration of the study.

### Data collection

**Fig. 1:**
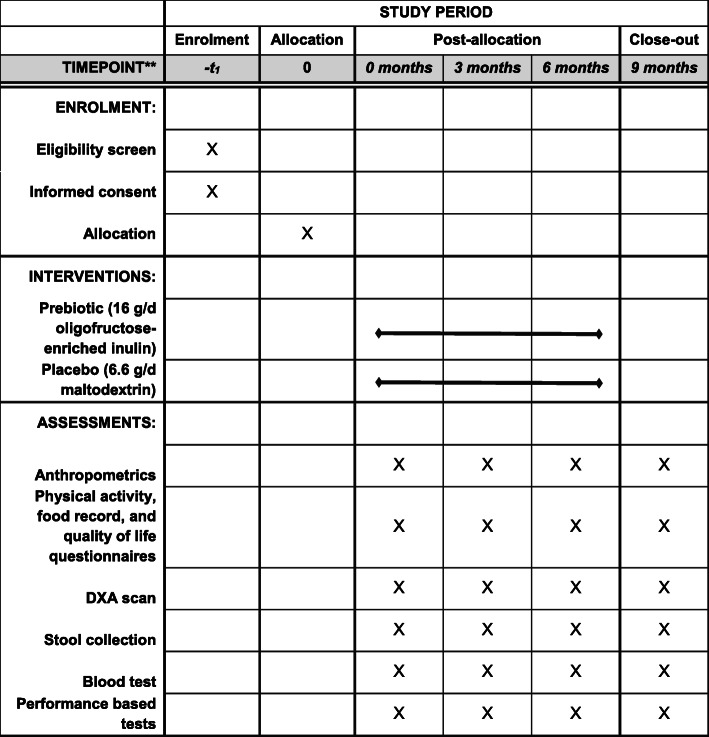
Schedule outlining the enrolment, interventions, and assessments for the proposed randomized controlled trial. Following eligibility assessment and informed consent collection (month 0), participants will be randomly allocated to either prebiotic (oligofructose-enriched inulin; 16 g/day) or placebo (maltodextrin; 6.6 g/day). Follow-up assessments will be performed at 3 and 6 months. After the 6 months’ intervention period, participants will stop taking study products and will be assessed again at 9 months to determine the persistence of the effects

The overall study design is depicted in Fig. 1. Participants will be assessed at the start of the diet intervention period (baseline = month 0), 3 months, 6 months (end of the diet intervention period), and 9 months (3-month wash out period following the end of the diet intervention). The primary outcome will be the change in performance-based tests in adults with obesity and knee osteoarthritis treated for 6 months with oligofructose-enriched inulin or placebo. Change in performance-based tests (30-s chair stand test, 40-m fast-paced walk test, time up & go, hand grip strength, and 6-min walk test) will be assessed by a subset of Osteoarthritis Research Society International (OARSI) tests for physical function in individuals with knee osteoarthritis. The secondary outcomes include change in knee pain, change in body composition (fat mass and lean mass), and change in quality of life in adults with obesity and knee osteoarthritis treated for 6 months with oligofructose-enriched inulin or placebo. To gain a deeper mechanistic understanding of the influence of the prebiotic on physical performance, gut microbiota composition, as well as serum inflammatory and metabolomics markers will also be assessed.

### Primary outcome measurements

#### Performance-based tests

1. The 30-s chair stand test is a test of lower body strength, endurance, and dynamic balance. Participants will be instructed, from a sitting position on a chair (44 cm seat height without arms), to stand up completely with fully extended hips and knees then completely return to the sitting position. This is repeated for 30 s and the number of repetitions is recorded [36–38].
2. The 40-m (4 × 10m) fast paced walk test is a test of short walking distance and changing direction while walking as fast as possible without running. Participants will be instructed to safely walk as fast as possible, along a 10-m walkway, and then turn around a cone, and return to the starting point and repeat again for a total of 40 m. The time of the test is recorded to the nearest 100th of a second and expressed as speed (m/s) by dividing the distance (40 m) by the time (s) it took to perform the test [38].
3. The time up & go is a transition test of ambulatory activity, including strength, agility, and dynamic balance. From a sitting position on a chair (44 cm seat height without arms), participants will be asked to stand up, walk to a cone 3 m away, turn around the cone, and return to sit back in the chair as fast and as safe as possible. Time is recorded in seconds to the nearest 10th of a second [37–39].
4. The 6-min walk test is a test of aerobic capacity and long distance walking activity. Participants will be instructed to walk as quickly as possible along a 40-m walkway, turn around a cone, return to the starting position, and repeat it again for 6 min to cover as much ground as possible. The distance walked over the 6 min is recorded in meters [37, 38, 40, 41].
5. The hand grip strength test is an objective measure of overall body muscle strength and physical function [42] as well as an important measure for frailty [43]. Participants will be asked to comfortably sit in a chair with both knees and hips flexed at 90°. Next, participants will hold the hand grip device (Jamar®, Patterson Medical, Warrenville, IL, USA) at 90° elbow flexion and be instructed to exhibit the greatest force possible, followed by a second trial with a 3-min rest interval between trials.

Improvement from baseline will be defined by an increase in the number of repetitions (30 s chair stand test), an increase in strength (hand grip strength), a reduction in the time to perform the 40-m fast-paced walk test, time up & go, or an increase in the distance covered for the 6-min walking test.

### Secondary outcome measurements

#### Outcomes related to knee joint pain

Numerical pain rating scale (NPRS) for knee pain: Participants will be asked to rate their pain intensity by selecting a number from 0 (no pain) to 10 (worst possible pain).

Knee injury and osteoarthritis outcomes score (KOOS): Participants will be asked to complete the self-administered KOOS questionnaire every 3 months, which consists of 5 subscales: pain, symptoms, function in activities of daily living, function in sports and recreation, and knee-related quality of life [44]. The questionnaire score ranges from 0 to 100, showing severe knee problems to no knee problems, respectively.

##### Pain medication questionnaire

At 0, 3, 6, and 9 months, participants will be asked to record knee pain medication name, dosage, and frequency over the past 7 days prior to coming to the test site.

#### Anthropometric measures

##### Anthropometrics

Current age, height, and sex will be collected. Weight will be measured using a calibrated balance beam scale at the start of the protocol to assess BMI and will be monitored every 3 months throughout the intervention period. Body composition will be assessed by dual energy x-ray absorptiometry (DXA; Hologic QDR 4500, Hologic, Inc., Bedford, MA).

##### Physical activity

Participants will be instructed to maintain their current level of physical activity throughout the study. Physical activity will be monitored every 3 months using a modified Godin’s leisure time exercise questionnaire, based on weekly exercise average intensity (strenuous, moderate, and mild) over the past month [45]. Furthermore, objective physical activity will be assessed by an accelerometer at the hip level at 30 Hz (ActiGraph Link®, Philips Healthcare; Andover, MA) for five consecutive days every 3 months. The metabolic equivalent (METs) will be calculated based on counts per minute. Counts per minute will be classified using already determined tri-axial vector magnitude cut-points for light, moderate, hard, or very hard using ActiLife6 software (ActiGraph, Pensacola, FL) [46].

#### Outcomes related to quality of life

Quality of life will be measured every 3 months using the SF-36 Health Survey questionnaire. This is a 36-question self-administered questionnaire to measure health on multi-dimensions, covering functional status, wellbeing, and overall evaluation of health [46]. Gastrointestinal feelings and bowel habits will be assessed every 3 months using our standard gastrointestinal feeling form that rates abdominal discomfort, bloating, flatulence, rumblings of stomach, and number of bowel movements [30].

### Food records

Participants will be encouraged to maintain their habitual diet intake throughout the study. Food and beverage will be assessed using a 3-day (two weekdays and one weekend day) food record every 3 months. This information will be analyzed with FoodWorks 18.0 software with the Canadian Nutrient File (The Nutrition Company, Long Valley, NJ) [31].

### Gut microbiota and inflammation related outcomes

Participants will be instructed on proper methods for stool sample collection and all materials provided in a convenient stool collection kit. Participants will collect 2 tablespoons of stool every 3 months. The container will be sealed, placed in a biohazard bag, transported to the lab, and stored at − 80 °C until processed.

#### Fecal microbiota analysis

Microbiota composition will be assessed as previously described [24, 47]. Bacterial genomic DNA will be extracted from stool samples using a fastDNA spin kit for feces (MP Biomedicals, Santa Anna, CA) and sequenced by the University of Calgary Centre for Health Genomics and Informatics using the Illumina 16S sequencing platform to amplify the V3-V4 region. Sequencing data from the MiSeq will be demultiplexed and converted to fastq format using Illumina’s bcl2fastq software. CutAdapt will be used to remove primers from reads and perform initial quality trimming. Following primer removal, length sequences of shorter than 10 bases pairs will be removed, and quality trimming threshold set at Q20. R package DADA2 will be used to infer amplicon sequence variants. Chimeric sequences will be removed, and taxonomic assignment performed using the RDP classifier and RDP database as reference.

#### Fecal short-chain fatty acid concentration

Reverse-phase high-performance liquid chromatography will be used as previously described to measure short-chain fatty acids, key metabolites produced by the gut microbiota, that act as signaling molecules to host tissues [48].

#### Serum endotoxin and inflammatory markers

Blood collected every 3 months by an experienced technician will be allowed to clot, serum collected, and then aliquots stored at − 80 °C until used. Serum will be used to measure endotoxin (lipopolysaccharide) using a PyroGene recombinant factor C endotoxin detection kit (Lonza, Walkersville, MD) [49]. Serum inflammatory biomarkers will be assessed using the 42-plex protein array panel at Eve Technologies (Calgary, AB) [50]. The 42-plex assay is a comprehensive panel of cytokines and chemokines that will help in elucidating the systemic effects of prebiotic exposure on systemic and local (knee joint) inflammation.

#### Serum metabolomics

Metabolomic profiles in serum will be assessed using proton nuclear magnetic resonance (^1^H NMR) spectroscopy as previously described [51, 52]. Blood metabolites can be useful in elucidating pathways by which interventions such as diet alter physiological outcomes [51, 52].

##### Compliance

Participants will be asked to return the product containers to assess compliance and actual product intake. Participants will purchase their own food and maintain their habitual food intake during the intervention period which will be monitored via completion of a 3-day food record every 3 months. Any changes or amendments to the protocol will be conveyed to the research ethics board via an official modification request and participants will be informed via electronic mail and/or phone.

### Sample size

Since there are no previous clinical trial studies assessing prebiotic supplementation in adults with co-morbid obesity and knee OA, the sample size calculation is based on data from relevant studies in obesity treatment. We have calculated sample size for both the primary and a secondary outcome. For the primary outcome, we used changes in isokinetic function for patients suffering from knee OA treated with an exercise intervention [53], showing that an *n* = 21 would provide 90% power at an alpha level of 0.05. For the secondary outcome, we used changes in body composition from a randomized placebo-controlled trial we conducted previously [25]. The calculation determined that an *n* = 23 would provide 90% power at an alpha level of 0.05 based on the decrease in fat mass in prebiotic versus placebo in adults with overweight or obesity. Given the length of the study (9 months) and the expected dropouts observed in intervention studies with dietary modification, additional participants will be added to account for potential dropouts. Therefore, *n* = 30 participants per treatment group (*n* = 60 total) will be recruited.

### Analysis

Statistical analysis will be performed using SPSS 26.0 software (IBM, New York, USA). Results will be considered statistically significant if *p* ≤ 0.05. Baseline descriptive data between the prebiotic and placebo groups will be compared using chi-square for categorical variables and t tests for continuous variables. The primary analysis will be on an intent-to-treat basis, with a secondary analysis performed on a per-protocol basis with all subjects that complete the intervention. The primary outcome measurement of performance-based tests will be expressed as mean with standard deviation and analyzed using a two-way mixed model ANOVA (2 × 4) with a within-subject factor (time) of 4 levels (0, 3, 6, and 9 month time points) and one between factor (group) of 2 levels (prebiotic and placebo). A similar statistical approach will be used to test differences in the secondary outcome measurements including DXA body scans, blood tests, stool collection, and questionnaires. For the microbiota analysis, Phyloseq R package will be used for downstream analysis. Alpha diversity will be measured by calculating the Shannon and Simpson index. Beta diversity will be evaluated using non-metric multidimensional scaling (NMDS) on Bray-Curtis dissimilarity matrix. Differential abundance analysis between groups will be carried out using LEfSe conda package. To account for multiple comparisons, a false discovery rate (FDR) correction will be applied and significance set at *p* ≤ 0.05.

All participant data will be coded with a study ID number. All data will be stored in a secure, locked office and password-protected computer. Data monitoring for safety is conducted through the Conjoint Health Ethics Research Board (University of Calgary). Interim analysis will occur when half of the participants have completed the intervention and study continuation decided by the steering committee. Auditing of the trial conduct will occur annually through the Conjoint Health Ethics Research Board. Participants will be informed of their rights to compensation in the consent form. The study findings will be disseminated to researchers through conference presentations and peer-reviewed publications and to the public and health care professionals through workshops and seminars. Additionally, the final results will be shared with the participants upon request via electronic mail, detailing which group the participant was enrolled as well as the changes for each variable from the start to finish of the study protocol.

## Discussion

The increased prevalence of adults with co-morbid obesity and knee OA has resulted in a growing demand for aggressive treatment strategies, such as bariatric surgery for substantial weight loss and/or knee joint replacement for pain management at increasingly younger ages. Despite a variety of factors that can play a substantial role in the development and further progression of OA, there is abundant evidence in the literature linking obesity with gut dysbiosis and increased low-level systemic inflammation, thereby contributing to early knee joint degeneration [6–10]. Currently, there is no curative treatment for OA, and patients have difficulties adhering to exercise treatment to improve knee joint function and reduce body weight due to discomfort and pain.

Since obesity is a known modifiable risk factor and there are few effective obesity treatments, a prebiotic diet intervention may offer a solution to improve quality of life in adults with obesity suffering from knee OA by reducing knee joint pain and improving physical function [28, 29]. Through positive changes in the gut microbiota, prebiotics have been shown to reduce low-level systemic inflammation and protect knee joint cartilage in rodent models of obesity [28, 29]. Based on the protective effects of prebiotics on joint health in rats and mice, and the reduction in inflammation in human studies, we hypothesize that prebiotic supplementation may be an effective, and most importantly, an easy to adhere conservative treatment that could significantly improve the quality of life of adults with obesity and concurrent metabolic knee OA. If proven to be successful, prebiotic supplementation could impact the health care system with reduced costs and potentially improved outcomes by targeting not only metabolic outcomes as previously shown with prebiotics [20–27], but also functional outcomes associated with less pain and improved knee function. Additionally, if effective, a regimen of prebiotic supplementation could provide a window of opportunity to motivate patients and improve their quality of life, whereby a reduction in knee joint pain through changes in gut microbiota could allow individuals to increase their physical activity or subsequently engage in an exercise program specifically aimed at restoring knee joint function due to OA. Lastly, positive findings from the present study could have the potential to postpone or potentially prevent the need for more aggressive treatment modalities for adults with obesity and concurrent knee OA, such as knee joint replacement.

This double-blind, placebo-controlled clinical trial is not without limitations and steps have been taken to reduce potential confounders and bias. First, this is an extensive and long-term study (9 months in total) and we speculate that participants may withdraw from the study. In order to accommodate for such losses, we will enroll 30% more participants than required on our sample size calculations. In some patients, oligofructose and inulin can cause result in increased flatulence and bloating. To minimize abdominal discomfort, participants will be instructed to start on a lower dose for the first 2 weeks to allow the digestive system to adjust to the increased intake of fermentable substrate. Finally, given the long duration of the study, patients might undergo lifestyles changes from the time of baseline assessments. To be able to monitor potential changes, dietary intake will be recorded with 3-day food records and physical activity monitored with self-report questionnaire and accelerometer results at 3 month intervals.

The work proposed here has the potential to identify a novel therapy that could alleviate some of the pressure on the growing demand for aggressive OA treatments such as total knee joint replacement at younger ages. Less aggressive, yet effective, conservative treatment options have the potential to address the growing prevalence of co-morbid obesity and knee OA by delaying the need for joint replacement or ideally preventing its need altogether. The results of this clinical trial will provide the first evidence regarding the effects of prebiotic supplementation on knee joint function and pain in adults with obesity who are also suffering from knee OA. If successful, the results may provide a simple, safe, effective, inexpensive, and easy to adhere intervention to reduce knee joint pain and improve the quality of life of adults with co-morbid knee OA and obesity.

## Trial status

The protocol (version 1.0, April 3, 2018) was approved by the Conjoint Health Research Ethics Board (REB17-2363), University of Calgary (Calgary, Alberta, Canada), on May 24, 2018, and was registered at ClinicalTrials.gov on 21 November 2019 (NCT04172688). Recruitment began in September 2018 and is expected to be completed by December 2021.

## Data Availability

Data is available for up to 5 years from the date of publication upon reasonable request.

## Abbreviations

OA: Osteoarthritis
SCFA: Short-chain fatty acids
HFS: High-fat/high-sucrose
BMI: Body mass index
DXA: Dual energy x-ray absorptiometry
OARSI: Osteoarthritis Research Society International
NPRS: Numerical pain rating scale
KOOS: Knee injury and osteoarthritis outcomes score
METs: Metabolic equivalent
NMR: Nuclear magnetic resonance
ANOVA: Analysis of variance
FDR: False discovery rate
NMDS: Non-metric multidimensional scaling
